# COMPAS-2: a dataset of *cata*-condensed hetero-polycyclic aromatic systems

**DOI:** 10.1038/s41597-024-02927-8

**Published:** 2024-01-19

**Authors:** Eduardo Mayo Yanes, Sabyasachi Chakraborty, Renana Gershoni-Poranne

**Affiliations:** Schulich Faculty of Chemistry, Technion - Israel Institute of Technology, Haifa, 32000 Israel

**Keywords:** Combinatorial libraries, Cheminformatics, Computational chemistry, Density functional theory

## Abstract

Polycyclic aromatic systems are highly important to numerous applications, in particular to organic electronics and optoelectronics. High-throughput screening and generative models that can help to identify new molecules to advance these technologies require large amounts of high-quality data, which is expensive to generate. In this report, we present the largest freely available dataset of geometries and properties of *cata*-condensed poly(hetero)cyclic aromatic molecules calculated to date. Our dataset contains ~500k molecules comprising 11 types of aromatic and antiaromatic building blocks calculated at the GFN1-xTB level and is representative of a highly diverse chemical space. We detail the structure enumeration process and the methods used to provide various electronic properties (including HOMO-LUMO gap, adiabatic ionization potential, and adiabatic electron affinity). Additionally, we benchmark against a ~50k dataset calculated at the CAM-B3LYP-D3BJ/def2-SVP level and develop a fitting scheme to correct the xTB values to higher accuracy. These new datasets represent the second installment in the COMputational database of Polycyclic Aromatic Systems (COMPAS) Project.

## Background & Summary

Polycyclic aromatic systems (PASs) are molecules composed of fused aromatic rings. They are an important and pervasive class of molecules, found in both the natural and man-made worlds, that has captivated researchers across many scientific disciplines, thanks to their remarkable structural and functional diversity. To date, PASs have been employed in a wide variety of uses, including as highly tunable fluorescent emitters^1–3^, catalysts^4,5^, organic semiconductors^6–8^, light-emitting diodes^9^, field effect transistors^10–12^, organic photovoltaics^13–15^, synthetic metals^16^, chemical sensors^17^, and even medicines^18,19^.

To design new molecules that can fully harness the potential functionality of PASs, it is necessary to understand their underlying structure-property relationships. However, due to the structural diversity of these compounds, uncovering such relationships is not straightforward. Data-driven approaches can accelerate the discovery and design of new functional molecules. Indeed, such approaches have already allowed the exploration of new swaths of chemical space^20–25^. However, the same tools have seen limited application for PASs^26–28^, because the large amounts of data that are necessary to apply them are not readily available. Indeed, despite the importance of PASs to many fields, this chemical space is under-represented in many existing databases, likely due to their molecular size and the computational cost associated with their characterization. Until recently, there were only a few examples of publicly accessible repositories containing appreciable numbers of polycyclic aromatic hydrocarbons (PAHs)^27,29,30^ and PASs^20^. Nevertheless, the more recently established OCELOT^31^ and PAH335^32^ datasets reiterate the importance of and the growing interest in this type of data.

To address the paucity of data for PASs in a methodical and organized manner, we conceptualized and initiated the COMPAS Project (COMputational database of PASs)^33^. Motivated by the understanding that big-data endeavors are crucial to guiding experimental efforts and advancing our chemical understanding^34^, we designed the COMPAS Project to enable data-driven investigations of PASs. Among the key features of the COMPAS database are: a) each dataset is generated at a uniform and suitable level of theory, which is necessary to allow the use of data-driven approaches and extraction of chemical insight; b) the data are curated and stored in a manner that is optimal for use with data science tools; c) inexpensive computational methods are benchmarked and fit to higher levels of accuracy, which enables rapid and affordable expansion of the database; d) all data is freely and openly accessible, in compliance with the FAIR principles^35^.

Herein, we present the second installment of the COMPAS Project, focused on *cata*-condensed heterocycle-containing PASs (cc-hPASs). Such molecules are especially promising as organic semiconductors^36–41^. We describe the construction of two datasets: COMPAS-2x and COMPAS-2D. The former contains the optimized geometries of 524,392 unique cc-hPASs calculated at the GFN1-xTB level^42^; the latter contains the optimized geometries of 52,000 cc-hPASs calculated at the CAM-B3LYP-D3BJ/def2-SVP level^43–48^. The molecules in both datasets range in size from 2 to 10 rings and are constructed from a library of 11 building blocks of diverse size, composition, and aromatic character. To our knowledge, these represent the largest and most structurally diverse datasets of cc-hPASs prepared to date. At the same time, we emphasize that the COMPAS Project is under constant expansion and future installments are already underway.

The current contribution joins the first installment, datasets COMPAS-1x and COMPAS-1D, which contain the structures and properties of *cata*-condensed polybenzenoid hydrocarbons (cc-PBHs) ranging in size from 1 to 11 rings (at the GFN2-xTB level) or from 1 to 10 rings (at the B3LYP-D3BJ/def2-SVP level), respectively^33^. These data can assist in guiding the synthesis of novel molecules, in screening for structures or substructures of interest, in probing fundamental properties (e.g., aromaticity, reactivity), or in training machine learning and deep learning models for various tasks. Indeed, we have recently reported on interpretable models for extracting chemical insight trained on COMPAS-1x^49,50^, as well as on a novel guided diffusion model for generating cc-hPASs with targeted properties, trained on some of the data described in this report^51^.

In the present report, we describe and discuss the following: a) the composition of the datasets; b) the workflow employed for data generation; c) benchmarking of the data against higher-level calculations and a fitting scheme for obtaining density functional theory (DFT)-level properties from GFN1-xTB calculations.

## Methods

In this section, we discuss our protocol for the enumeration of a random subset of the chemical space of *cata*-condensed heterocycle-containing PASs (cc-hPASs) and the high-throughput computations employed to obtain optimized geometries and molecular properties with different methods.

### Building-block library

**Fig. 1:**
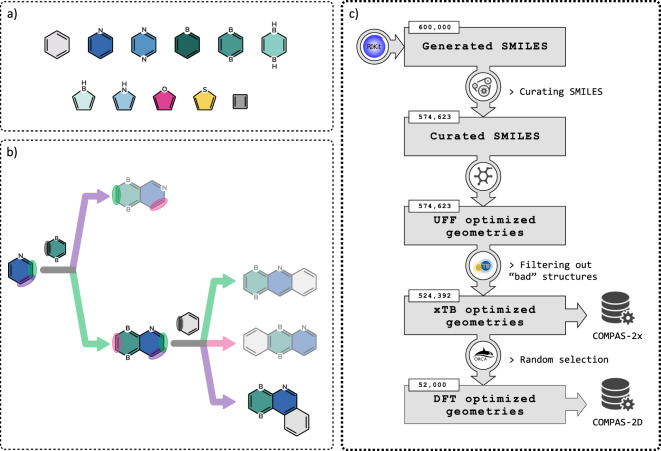
Various aspects of the COMPAS-2 generation protocol: (**a**) Library of cyclic building blocks used in COMPAS-2; (**b**) An example of an enumeration pathway for generating a tricyclic cc-hPAS molecule; (**c**) The data generation workflow, from structure enumeration to high-throughput calculations to obtain optimized structures and molecular properties.

For the construction of the cc-hPAS molecules in COMPAS-2, we used a library of 11 cyclic building blocks, varying in size (from four- to six-membered rings), composition (B, N, O, and S mono- and di-substitution), and aromatic character (aromatic and antiaromatic). Namely, these building blocks are: benzene, pyridine, pyrazine, borinine, 1,4-diborinine, 1,4-dihydro-1,4-diborinine, borole, pyrrole, furan, thiophene, and cyclobutadiene (shown in Fig. 1a). These specific moieties were chosen due to their prevalence and importance in various functional PASs, in particular in the field of organic electronics^36,39–41^. The number of building blocks was limited to 11, which allows us to sample a broad diversity of structures and properties within a feasible number of molecules.

### Enumeration Protocol

To generate cc-hPASs from the building blocks detailed in Fig. 1a, we designed and implemented an enumeration pipeline using SMARTS (SMILES arbitrary target specification language^52^; SMILES - Simplified Molecular Input Line Entry System)^53,54^. Using the SMARTS representation, we encoded different ‘reactions’ of fusing two rings together in a *cata*-condensed fashion (*cata*-condensation refers to a manner of ring fusion whereby each atom is shared by, at most, two rings). Each ‘reaction’ creates a fused bond that is shared by two adjoining rings, i.e., two neighboring atoms that are endocyclic to both rings (see Section S2 in the Supporting Information for further details on the SMARTS representation). By performing sequential ‘reactions’, we generated 600,000 polycyclic compounds.

In generating the structures, we imposed several (arbitrary) constraints. First, to simplify the generation rules and the resulting structures, we opted to allow only carbon atoms on the fused bonds. In other words, heterocyclic moieties can only fuse at their C-C bonds; heteroatoms remain on unfused bonds (the SMARTS formalism for this ‘reaction’ is shown in Table 1). Second, to ensure only *cata*-condensation is achieved, both carbons in the C-C bond chosen for fusion must belong to only one ring of the nascent cc-hPAS. Third, in cases where more than one C-C bond is suitable for fusion, the choice of which bond to use as the fusion site is random. Fourth, in the ring choice step, we invoked a bias of 10:1 favoring benzene over all other rings (see Section S2 in the Supporting Information for further details). This was done to ensure a more realistic distribution of structures. Fifth, we biased the generation towards molecules of intermediate size (8 and 9 rings) and limited the size of generated molecules to 10-ring systems. The rationale behind this choice was that these sizes provide large structural diversity, and any structure-property relationships should already become obvious in systems of this size (as we previously showed for the cc-PBHs)^33,49,50^. Thus, there was no need to perform calculations of larger systems, which would be substantially more resource-consuming.

**Table 1:** Table of fragments and their SMARTS encodings.

Fragment attached	SMARTS encoding
Benzene	[#6;R1:1]~[#6;R1:2]»[c:2]:1:[c:1]:[c:3]:[c:4]:[c:5]:[c:6]:1
Pyridine	[#6;R1:1]~[#6;R1:2]»[c:6]:1:[c:2]:[c:1]:[n:3]:[c:4]:[c:5]:1
Borinine	[#6;R1:1]~[#6;R1:2]»[#5;a:3]:1:[c:1]:[c:2]:[#6;a:6]:[c:5]:[c:4]:1
Pyrazine	[#6;R1:1]~[#6;R1:2]»[c:2]:1:[c:1]:[n:3]:[c:4]:[c:5]:[n:6]:1
1,4-diborinine	[#6;R1:1]~[#6;R1:2]»[#5;H0;a:6]:1:[#6:2]:[#6:1]:[#5;H0;a:3]:[#6:4]:[#6:5]:1
1,4-dihydro-1, 4-diborinine	[#6;R1:1]~[#6;R1:2]»[H][#5:3]-1-[c:1][c:2]-[#5:6]([H])-[c:5][c:4]-1
Pyrrole	[#6;R1:1]~[#6;R1:2]»[c:2]:1:[c:5]:[c:4]:[n:3]([H]):[c:1]:1
Borole	[#6;R1:1]~[#6;R1:2]»[c:2]:1:[c:5]:[c:4]:[b:3]([H]):[c:1]:1
Thiophene	[#6;R1:1]~[#6;R1:2]»[c:2]:1:[c:5]:[c:4]:[s:3]:[c:1]:1
Furan	[#6;R1:1]~[#6;R1:2]»[c:2]:1:[c:5]:[c:4]:[o:3]:[c:1]:1
Cyclobutadiene	[#6;R1:1]~[#6;R1:2]»[c:1]1[c:2][c:4][c:3]1

The structure generation workflow consists of the following steps:

- Step 1: Generate a random integer (*n*) between 1 and 10.
- Step 2: Randomly select *n* building blocks from the library and store them in a list. The order of the list will be the order of addition of the building blocks to the nascent molecule.
- Step 3: Initialize the nascent molecule with the first building block in the list.
- Step 4: Join the next building block in the list to each of the available C-C bonds in the molecule in turn, each time creating a new structure.
- Step 5: Check all of the resulting structures for chemical validity. Correct errors (e.g., double-bond placement) and remove duplicates.
- Step 6: Randomly select one of the structures. This is now the nascent molecule.
- Step 7: Repeat steps 4–6 until all building blocks in the list have been added.

Figure 1b presents a schematic illustration of an enumeration process leading to a tricyclic product. In the scheme, we show all of the possible resulting structures (which are constitutional isomers), however, in practice, a deterministic choice was made at each step, leading to a single product at the end of each enumeration process. In our example, the process began by randomly choosing *n* = 3 and a list of building blocks comprising pyridine, 1,4-diborinine, and benzene (in that order). In principle, pyridine (shown in blue) has four C-C bonds that can serve as fusion sites. However, only two of them are unique, due to the symmetry of the molecule (these are circled in purple and green, respectively). The next building block that was randomly chosen was 1,4-diborinine (shown in turquoise), which has only one type of fusion site (circled in gray). Joining this new building block to the nascent molecule (pyridine) at the bond circled in purple led to the bicyclic product shown on top (following the purple arrow); joining the new building block at the C-C bond circled in green led to the bottom bicyclic product (following the green arrow). The algorithm then randomly chose to continue with the bottom product (hence, the top one is faded out and there was no continuation of molecular construction). This nascent molecule had three potential fusion sites (circled in pink, green, and purple, respectively). Following the similarly-colored arrows led to each of the three tricyclic products that were obtained through the fusion of the third building block, benzene (shown in gray). At this point, the algorithm once again randomly selected only one of the products (in this case, the bottom one; the other two are faded out). Having reached the end of the building block list, the algorithm recognized that the construction had been completed and entered the selected molecule into the dataset. All other structures generated in the process were discarded.

**Fig. 2:**
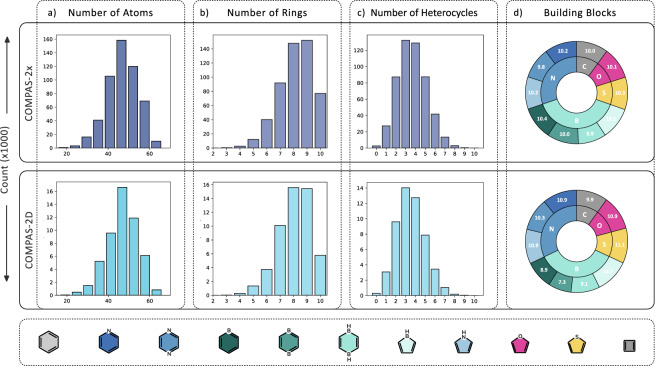
Overview of the data distribution in the COMPAS-2 datasets: top: COMPAS-2x; bottom: COMPAS-2D. (**a**) histogram of the number of atoms in each molecule; (**b**) histogram of the number of rings in each molecule; (**c**) histogram of the number of heterocycles contained in each molecule; (**d**) doughnut charts of the frequency of different atoms (inner ring) and building blocks (outer rings) present in the dataset. The corresponding color-coded legend of the individual building blocks is also provided.

We performed this generation process 600k times and, following each generation process, the resulting cc-hPAS was annotated with its canonical SMILES and InChI^55,56^ representations using RDKit^57^. The InChI representation was used to identify and remove duplicate entries. We note that the current enumeration protocol is not memory efficient and may be improved using graph-theoretical methods. Nevertheless, it ensures an exhaustive exploration of the constitutional isomer chemical space (within the described constraints) and generates unique cc-hPASs. The histograms of the various structural features present in the dataset (Fig. 2) show that the distribution of molecular sizes and compositions is well sampled.

### High-throughput data generation

Using the protocol described above, we enumerated the InChI representations for a diverse set of 600,000 cc-hPASs. These molecules were then put through a high-throughput computational pipeline to obtain optimized geometries and molecular properties. The steps of the workflow (shown schematically in Fig. 1c) are as follows:

- Step 1: Embed the molecule in 3D space using the Experimental-Torsion Distance Geometry (ETDKG) method with additional “basic knowledge”^58,59^, as implemented in RDKit.
- Step 2: Pre-optimize the structure with the universal force field (UFF)^60^, as implemented in RDKit. (We note that because sp^3^ hybridized B parameters are unavailable in UFF (RDKit), we used sp^2^ B parameters. Although this led to suboptimal pre-optimized structures, the subsequent steps ensured close approximations to the ground truth.)
- Step 3: Optimize the structure at the GFN1-xTB level using the xTB software^61^ (see Section S3 in the Supporting Information for further details on benchmarking and choice of method).
- Step 4: Calculate harmonic vibrational frequencies to ensure the geometry is a minimum on the potential energy surface (i.e., *N*_*imag*_ = 0).
- Step 5: Filter out molecules that did not optimize correctly (i.e., optimization did not converge, presence of imaginary frequencies, presence of bond lengths greater than 2.0 Å, or presence of atom-atom distances shorter than 0.1 Å.)
- Step 6: If the obtained structure passes the validity check, optimize the geometries and calculate the frequencies of the anionic and cationic forms of the molecule at the GFN1-xTB level.
- Step 7: Repeat Step 5 for the cationic and anionic forms.

With this pipeline, we obtained the optimized geometries and molecular properties of 524,392 cc-hPASs (corresponding to 22,735 molecular formulae), calculated at the GFN1-xTB level–these comprise the COMPAS-2x dataset. Figure 2a shows the structural diversity of the molecules contained in the COMPAS-2x dataset in terms of molecular size and the distribution of heterocyclic moieties among the molecules.

We note that the majority of COMPAS-2x molecules are medium-sized molecules with ~50 atoms, comprising 8 or 9 rings (Fig. 2a,b, top row). This is because we used a quasi-Poisson distribution to bias the size of the generated molecules towards 8 and 9 rings. Hence, the 10-ring family is smaller even though the number of possible structures increases substantially with the increase in the number of rings. We observe a distribution of the number of heterocycles per molecule, with most of the compounds containing 3 or 4 heterocycles (Fig. 2c, top row). The distribution of the different heterocycles is uniform (Fig. 2d, top row). Overall, the histograms show that the enumeration protocol successfully generates a random and broad sampling of the chemical space.

From COMPAS-2x, we randomly chose 52,000 molecules (approximately 10%), for which we performed geometry optimizations and property calculations with density functional theory (DFT) using the ORCA software^62,63^. For these calculations, we employed the CAM-B3LYP functional^43^ with the def2-SVP basis set^47^, using Grimme’s D3 dispersion correction^44,45^ with Becke-Johnson damping^46^. The DFT-optimized geometries and molecular properties of these 52,000 cc-hPASs comprise the COMPAS-2D dataset (corresponding to 9,776 molecular formulae). The assessment of structural diversity for the COMPAS-2D dataset (Fig. 2, bottom row) shows that the distribution of COMPAS-2D is similar to that of COMPAS-2x, indicating that the selection was successfully random and that this dataset is a good sampling of the chemical space, as well.

## Data Records

The COMPAS Project is hosted on the Poranne Group’s GitLab repository (https://gitlab.com/porannegroup/compas) and is openly and freely available. A minted version of the data reported in this manuscript is available on Figshare (10.6084/m9.figshare.24347152) (dataset posted on 2023-10-19)^64^. Furthermore, a website (https://compas.net.technion.ac.il/) has been developed and deployed to facilitate sub-structure and property-based queries. The current contribution expands the existing database with two datasets: COMPAS-2x (524,392 cc-hPASs; geometries and properties calculated with GFN1-xTB) and COMPAS-2D (52,000 cc-hPASs; geometries and properties calculated at the CAM-B3LYP-D3BJ/def2-SVP level). Additionally, we include in COMPAS-2x properties of the neutral compounds in COMPAS-2x, which have been corrected from the GFN1-xTB to the CAM-B3LYP-D3BJ/def2-SVP level, using a multi-linear regression correction scheme (see below for further details). Jupyter notebooks used to perform data analyses and multi-linear regressions are also available on the GitLab repository.

### File Format

All molecular geometries optimized at the GFN1-xTB and CAM-B3LYP-D3BJ/def2-SVP levels are publicly available for download as compressed sdf files *COMPAS-2x.sdf.gz* and *COMPAS-2D.sdf.gz* files, respectively, from https://gitlab.com/porannegroup/compas. These files contain the optimized geometries (Cartesian coordinates and connectivity information) of 524,392 and 52,000 molecules, respectively. All molecular properties computed at the GFN1-xTB and CAM-B3LYP-D3BJ/def2-SVP level for these optimized geometries in their neutral, cationic, and anionic forms are publicly available for download as *COMPAS-2x.csv*, and *COMPAS-2D.csv* files, respectively, from https://gitlab.com/porannegroup/compas.

### Properties

The columns of the *.csv* files correspond to the properties described in Table 2. For every molecule in COMPAS-2x and COMPAS-2D, the respective dataset contains the molecular formula, number of atoms, types of atoms, InChI, SMILES, charge, energy of the highest occupied molecular orbital (HOMO), energy of HOMO–1, energy of the lowest unoccupied molecular orbital (LUMO), energy of LUMO + 1, energy of the HOMO-LUMO gap (Gap, Eq. 1), adiabatic ionization potential (AIP, Eq. 2), and adiabatic electron affinity (AEA, Eq. 3), along with several structural properties, as listed in Table 2. In addition, COMPAS-2x contains the zero-point correction to the energy (ZPE) and the total GFN1-xTB energy (E_tot_(xTB)), which is the sum of the electronic energy calculated with the self-consistent-charge method (this includes the D4 dispersion correction). COMPAS-2D contains the DFT total energy (E_tot_(DFT)), which is the sum of the self-consistent field electronic energy, the nuclear repulsion, and the D3-BJ dispersion correction. *COMPAS-2x.csv* also contains “corrected” properties for the neutral state of the 524,392 molecules in COMPAS-2x; i.e., these are values that have been linearly regressed with respect to the *COMPAS-2D.csv* (corrected from xTB to DFT level). The input templates used to compute the properties for COMPAS-2x and COMPAS-2D are provided in Section S1 of the Supporting Information.

**Table 2:** Property keys, units of the respective quantities, and description of the molecular data present in *COMPAS-2x.csv* and *COMPAS-2D.csv* files.

Properties	Units	Description	COMPAS-2x	COMPAS-2D
name	—	9-character alpha-numeric name	✓	✓
charge	e	Charge on the molecule	✓	✓
formula	—	Molecular formula	✓	✓
inchi	—	InChI descriptor	✓	✓
smiles	—	SMILES descriptor	✓	✓
rings	—	Number of rings	✓	✓
aromatic_rings	—	Number of aromatic rings	✓	✓
atoms	—	Total number of atoms	✓	✓
heteroatoms	—	Number of heteroatoms	✓	✓
heterocycles	—	Number of heterocycles	✓	✓
branch	—	Number of branches	✓	✓
cyclobutadiene	—	Number of cyclobutadiene rings	✓	✓
pyrrole	—	Number of pyrrole rings	✓	✓
borole	—	Number of borole rings	✓	✓
furan	—	Number of furan rings	✓	✓
thiophene	—	Number of thiophene rings	✓	✓
dhdiborinine	—	Number of 1,4-dihydro-1,4-diborinine rings	✓	✓
14diborinine	—	Number of 1,4-diborinine rings	✓	✓
pyrazine	—	Number of pyrazine rings	✓	✓
pyridine	—	Number of pyridine rings	✓	✓
borinine	—	Number of borinine rings	✓	✓
benzene	—	Number of benzene rings	✓	✓
h	—	Number of hydrogen atoms	✓	✓
c	—	Number of carbon atoms	✓	✓
b	—	Number of boron atoms	✓	✓
s	—	Number of sulfur atoms	✓	✓
o	—	Number of oxygen atoms	✓	✓
n	—	Number of nitrogen atoms	✓	✓
homo	eV	Energy of the HOMO	✓	✓
lumo	eV	Energy of the LUMO	✓	✓
lumo + 1	eV	Energy of the LUMO + 1	✓	✓
homo - 1	eV	Energy of the HOMO - 1	✓	✓
gap	eV	Energy of the LUMO − Energy of the HOMO	✓	✓
nfod	—	Fractional occupation density	✓	
zero_point_energy	Eh	Zero point energy of molecule	✓	
dispersion	Eh	Dispersion correction	✓	✓
energy	Eh	Final energy of molecule	✓	✓
aip	eV	Adiabatic ionization potential	✓	✓
aea	eV	Adiabatic electron affinity	✓	✓
dipole	D/a.u.	Dipole vector of the molecule	✓	✓
homo_corr	eV	xTB-level HOMO corrected to DFT-level	✓	
lumo_corr	eV	xTB-level LUMO corrected to DFT-level	✓	
gap_corr	eV	xTB-level Gap corrected to DFT-level	✓	
energy_corr	Eh	xTB-level E_*tot*_ corrected to DFT-level	✓	
aip_corr	eV	xTB-level AIP corrected to DFT-level	✓	
aea_corr	eV	xTB-level AEA corrected to DFT-level	✓	
rmsd	Å	Root mean square deviation between xTB- and DFT-optimized structures		✓

HOMO, LUMO, Gap, AIP, and AEA are provided only for neutral systems. Energies are provided in electron volts (eV) or Hartree (Eh) units. Charge is reported in atomic units (a.u.). The dipole vector for COMPAS-2x is in Debye (D) and in atomic units (a.u.) for COMPAS-2D.

The Gap, AIP, and AEA are calculated as follows:

1 $${\rm{Gap}}={\rm{LUMO}}-{\rm{HOMO}}$$

2 $${\rm{AIP}}={{\rm{E}}}_{{\rm{tot}}}\,{}^{{\rm{cation}}}-{{\rm{E}}}_{{\rm{tot}}}\,{}^{{\rm{neutral}}}$$

2 $${\rm{A}}{\rm{E}}{\rm{A}}={{\rm{E}}}_{{\rm{t}}{\rm{o}}{\rm{t}}}{}^{{\rm{a}}{\rm{n}}{\rm{i}}{\rm{o}}{\rm{n}}}-{{\rm{E}}}_{{\rm{t}}{\rm{o}}{\rm{t}}}{}^{{\rm{n}}{\rm{e}}{\rm{u}}{\rm{t}}{\rm{r}}{\rm{a}}{\rm{l}}}$$

where E_tot_^neutral^ is the dispersion-corrected total energy of the optimized molecule in the neutral form, and E_tot_^cation^ and E_tot_^anion^ are the dispersion-corrected total energies of the molecule in the cationic and anionic forms, respectively.

## Technical Validation

### Comparison between GFN1-xTB and CAM-B3LYP-D3BJ results

The speed and low computational cost of GFN1-xTB make it ideal for high-throughput exploration of large chemical spaces. Naturally, this comes at the expense of accuracy; although xTB is considered to give good energies for reactions, as a semi-empirical method it is less accurate than higher-level *ab initio* and most modern DFT methods. Nevertheless, it is possible to leverage the advantages of the less expensive calculations if a robust scheme can be constructed to correct the GFN1-xTB calculated properties towards a higher accuracy level. In this section, we compare the results obtained with GFN1-xTB to those obtained with CAM-B3LYP-D3BJ/def2-SVP and implement such a correction scheme.

**Fig. 3:**
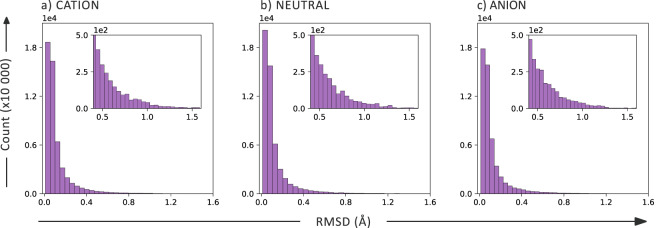
RMSD (Å) between the structures optimized at GFN1-xTB and CAM-B3LYP/def2-SVP level of theory for the: (**a**) Cation, (**b**) Neutral, and (**c**) Anion species.

We first compare the geometries obtained by the two levels of theory. The root mean square deviations (RMSDs) for the cationic, neutral, and anionic species are shown in Fig. 3. We observe that the agreement between the geometries obtained with the two methods is satisfactory, with an RMSD < 0.2 Å for 83%, 88%, and 87% of the cationic, neutral, and anionic molecules, respectively. This demonstrates that the xTB method manages to provide similar geometries to the more expensive DFT method. Moreover, it shows that the charge of the species does not affect the agreement between the methods.

**Fig. 4:**
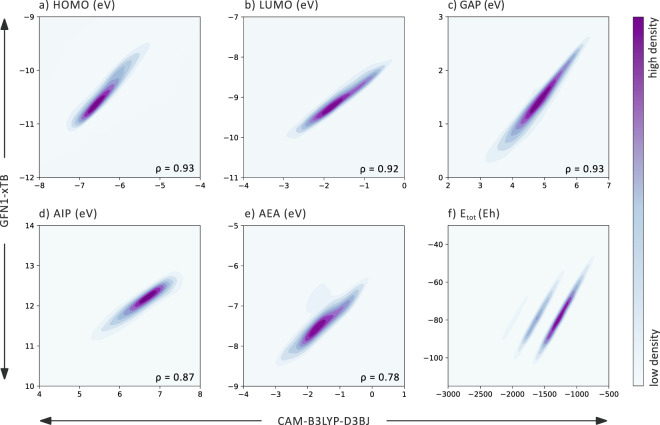
Comparison of GFN1-xTB and CAM-B3LYP-D3BJ/def2-SVP calculated values for: (**a**) HOMO (eV), (**b**) LUMO (eV), (**c**) Gap (eV), (**d**) AIP (eV), (**e**) AEA (eV), and (**f**) E_tot_ (Eh). The colors of the contour plots indicate the density of points in the region: darker shades indicate high density, lighter shades indicate low density.

Next, we compare the values of various properties in the datasets. Figure 4 displays contour plots of the HOMO, LUMO, Gap, AIP, AEA, and E_tot_ data calculated with the two methods. From the axis values, it is immediately apparent that the values calculated with GFN1-xTB span an entirely different range than those calculated with DFT. For DFT, the orbital energy values are almost always less negative (for HOMO by 4 eV, for LUMO by 7 eV, and accordingly for Gap by 3 eV. For AIP, DFT values are 5 eV lower, consistent with a less negative HOMO. Similarly, for AEA, DFT gives values that are 5 eV less negative, which agrees with a higher-lying LUMO. Despite the offsets in the property value ranges, the plots of the data show that the two methods are reasonably well correlated. The Pearson correlation coefficients (noted for plots (a–e) on each respective plot) range from 0.78 to 0.93, implying that the individual correlations are largely linear. Taking into account only the 80% of the data present in the densest regions increases the coefficients to 0.93–0.97 (see Section S4 in the Supporting Information for scatter plots of the data). We note that the agreement for the AEA is the poorest of all the properties, just as it was for the cc-PBHs we studied previously^33^. We hypothesize that this is because the basis set used for the DFT calculations (def2-SVP, which does not contain diffuse functions) is not optimal for anionic systems, especially non-planar ones. Despite this, the overall satisfactory agreement suggests that the choice of this inexpensive basis set is justified. An interesting phenomenon is observed in the plot of the E_tot_ (Fig. 4f): a series of separate linear correlations is obtained, rather than one main grouping. By examining the structural features of the molecules contained within each grouping, we determined that the differentiation stems from the number of sulfur atoms in the molecule (for further information, see Section S5 in the Supporting Information). We note that a similar issue was reported for organosilicon compounds^65^, indicating that there may be a general discrepancy between xTB and DFT in treating third-period atoms. This means that any correction scheme must take this structural information into account. Nevertheless, the remarkably good linear correlation between the two methods suggests that a suitable regression can be constructed to correct this behavior.

### GFN1-xTB Corrected Towards CAM-B3LYP-D3BJ

The high Pearson coefficients of the scatter plots (Fig. 4) suggest that linear regressions may be sufficient to correct the GFN1-xTB data towards the CAM-B3LYP-D3BJ/def2-SVP level. Hence, we employed a multi-linear regression, using the GFN1-xTB calculated property value as the baseline and the molecular formula of the molecule as the feature set (for further details see Section S6 of the Supporting Information, which also describes additional regression models that were tested). The advantage of this model is its simplicity–it does not require any knowledge of the specific molecular structure beyond the atomic composition. This model is reminiscent of the quasi-atom corrections^66–68^ often used in correcting DFT-level properties, such as formation enthalpies, with respect to composite wavefunction theories^69^.

We used the COMPAS-2D molecules as the benchmarking dataset and extracted the property values of the same 52,000 molecules from the COMPAS-2x dataset. We then separated the 52,000 molecules into training (80%) and test (20%) sets and used the training set to optimize the coefficients of the multi-linear regression for each property with respect to the individual features (i.e., numbers of atoms of each type). The coefficients and intercepts obtained from the multi-linear regression are detailed in Table 3. We specifically note the anomalously high coefficient for sulfur atoms in the regression for E_tot_, which relates to our previous observation regarding the dependence of the energy on the number of sulfurs, as described above.

**Table 3:** Statistical data for correction schemes from xTB-calculated properties to DFT-level properties, for the COMPAS-2D molecules.

Property	H	C	B	S	O	N	xTB	Intercept	R^2^	RMSE	MAE
HOMO	0.0124	0.0065	−0.0423	−0.0370	0.0258	−0.0253	1.2397	6.2841	0.89	0.16	0.11
LUMO	0.0160	−0.0079	−0.0862	−0.0303	0.0238	−0.0093	1.0648	8.1821	0.94	0.14	0.10
Gap	0.0263	−0.0245	−0.0358	0.0222	0.0190	0.0331	1.1815	3.4427	0.89	0.23	0.16
AIP	0.0061	0.0088	0.0608	0.0824	0.0042	0.0538	1.3537	−10.3931	0.84	0.22	0.14
AEA	0.0504	−0.0103	−0.1113	0.0012	0.0469	0.0164	0.8820	4.6421	0.74	0.32	0.23
E_tot_	−0.2102	−36.3793	−23.6932	−395.1947	−71.4597	−52.1923	0.7880	0.1285	1.00	0.02	0.02

For all multi-linear regressions, the coefficients of atomic features, R^2^, RMSE, and MAE are reported. RMSEs and MAEs for all properties are reported in eV, except for E_tot_, which is reported in Eh.

The resulting fitting equations were then used to correct the GFN1-xTB calculated properties of the test set. The agreement between the values predicted by the corrected scheme and the DFT-calculated values was evaluated (Table 3). Remarkably good correlations were obtained with this very simple regression method and the mean absolute errors (MAEs) indicate that the properties are calculated with satisfying accuracy (especially considering the low computational cost): 0.11 eV, 0.10 eV, 0.16 eV, 0.14 eV, 0.23 eV, and 0.02 Eh for the HOMO, LUMO, Gap, AIP, AEA, and E_tot_, respectively. The coefficients of determination (ranging between 0.74–1.00) indicate a high measure of linear correlation and good prediction performance. We note that the property with the highest error is the AEA. This is not surprising, given that this property also showed the lowest linearity in Fig. 4e, as we discussed above. Although slightly better agreements can be achieved with more sophisticated models (see Section S6 in the Supporting Information), the simplicity, transparency, and interpretability of the multi-linear regression make it an attractive choice.

**Fig. 5:**
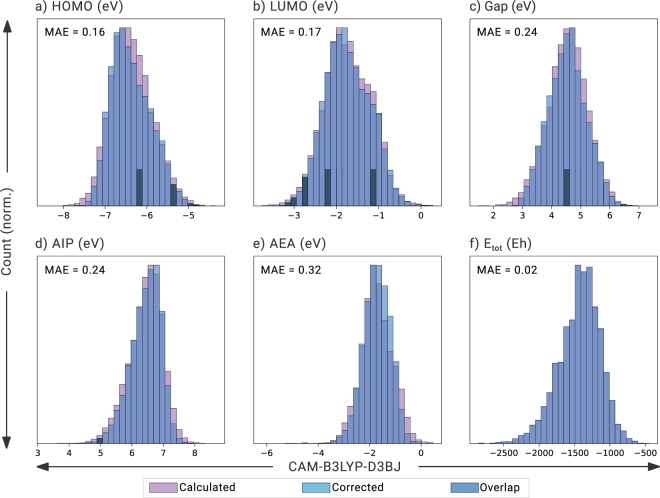
Comparison of electronic properties obtained with the multi-linear correction scheme (Corrected) against DFT-calculated properties (Calculated) on the test set (20% of COMPAS-2D).

To evaluate the performance of our correction scheme, we plotted superimposed histograms of the corrected properties and the DFT-calculated properties for the molecules in our test set (20% of the molecules in COMPAS-2D, which were not used in training the models, *vide supra*). These histograms are shown in Fig. 5 (the values predicted with our correction scheme are shown in light blue; the values calculated for the same molecules with DFT are shown in darker blue). Across all properties, we note a very high degree of overlap, suggesting that the models capture not only the average values (see Table 3), but also the distribution of the data well, and our correction scheme is therefore transferable to other cc-hPASs. This allows us to generate additional datasets with rapid and inexpensive calculations, and easily correct the values towards the more expensive and more accurate DFT level (as we have already done for COMPAS-2x). Although higher accuracy is not always necessary to gain insight and learn structure-property relationships (as we have recently shown^51^), it can be crucial in certain cases. For example, for calculation of properties such as oxidation potential and power conversion efficiency, which are parameterized against experimentally obtained reference states.

In summary, the COMPAS-2 datasets represent the largest freely available dataset of PASs to date. As the second installment in the still-growing COMPAS Project, COMPAS-2 promises discoveries of hitherto unknown chemical trends in the cc-hPAS chemical space. Considering the importance and prevalence of PASs in chemistry and materials science, these new data can be used to advance a wide variety of disciplines with new opportunities for data-driven investigations to enable the identification of novel functional molecules that may find applications in organic semiconductors and optoelectronics.

## Usage Notes

The Python 3.10^70^ code used to enumerate the molecular structures, to perform geometry optimization, and to analyze the data are available on GitLab. The repository contains several Python scripts and Jupyter notebooks:

- Scripts to generate molecular structures.
- Scripts to perform semi-empirical calculations and compile the results.
- Scripts to perform DFT calculations and compile the results.
- Notebooks to walk through the chemical library enumeration, data curation, and annotation and reproduce the figures.
- Notebooks to perform the xTB-to-DFT correction.

### Supplementary information

Supplementary Information

## Data Availability

All code is available on the Poranne Group repository on GitLab: https://gitlab.com/porannegroup/compas, licensed under a CC-BY license. Further details are provided in the repository’s online README.md file.
