# Supplementary material for: COMPAS-2: a dataset of cata-condensed hetero-polycyclic aromatic systems
Source: Sci Data. 2024 Jan 19;11:97. doi: 10.1038/s41597-024-02927-8 (PMC10799083; doi:10.1038/s41597-024-02927-8)
Supplement: Supplementary file 1 — Supplementary Information [file 41597_2024_2927_MOESM1_ESM.pdf]

Supporting Information

COMPAS-2: a dataset of *cata*-condensed hetero-polycyclic aromatic systems

Eduardo Mayo Yanes,[a] Sabyasachi Chakraborty,[a] and Renana Gershoni-Poranne*[a]

[a] *Schulich Faculty of Chemistry, Technion – Israel Institute of Technology, Haifa 32000, Israel*

e-mail: rporanne@technion.ac.il

Contents

S1 Computational Details **S3**
S1.1 Software Versions S3
S1.2 Calculation Input Templates S3
S1.2.1 Coordinates embedding and UFF optimization S3
S1.2.2 GFN1-xTB and GFN2-xTB calculations S3
S1.2.3 MOPAC2016 calculations S4
S1.2.4 DFT calculations S5

S2 Structural descriptors and moiety distribution **S6**

S3 Benchmarking Semi-empirical Methods **S7**

S4 Scatter Plots of GFN1-xTB versus DFT-Calculated Properties **S9**

S5 The Effect of S on total energy **S10**
S5.1 Linear Regression Between GFN1-xTB and CAM-B3LYP-D3BJ . . . S11

S6 Benchmarking Regression Schemes **S12**

List of Tables

S1 SMARTS patterns for aromatic moieties. S6
S2 Building block distribution in COMPAS-2x and COMPAS-2D S6
S3 Correlation of HOMO and LUMO calculated by DFT and semi-empirical methods. S8
S4 Error metrics for different regression models. S13

List of Figures

S1 HOMO & LUMO energies calculated using semiempirical and DFT methods. S7
S2 Comparison of electronic properties calculated at the xTB and DFT levels of theory S9
S3 Influence of S on energies. S10
S4 Linear regression of GFN1-xTB energies using atom counts. S11

S1 Computational Details

S1.1 Software Versions

The following software packages were used in the course of this work:

1. For enumeration and descriptor calculations - RDKit (v2022.03)[1] and Python (v3.9),[2] including libraries provided by these packages.

2. For GFN1-xTB and GFN2-xTB calculations - xTB (v6.5.1.27).[3–5]

3. For PM6 and PM7 calculations - MOPAC2016.[6,7]

4. For DFT calculations - ORCA (v5.2.6).[8,9]

S1.2 Calculation Input Templates

In this section, we provide templates for all types of calculations performed in the course of this work.

S1.2.1 Coordinates embedding and UFF optimization

The following Python functions provide the input templates for coordinates embedding and Univeral Force Field (UFF) optimization[10] using RDKit:

```
def gen3D(mol):
    try:
        embedded = AllChem.EmbedMolecule(mol, ETversion=2)
    except:
        embedded = -1
    return mol, embedded

def uff_specialoptimize(mol):
    spboron_pattern = Chem.MolFromSmarts("[#5H0]")
    print(mol.GetSubstructMatches(spboron_pattern))
    for match in mol.GetSubstructMatches(spboron_pattern):
        boron = mol.GetAtomWithIdx(match[0])
        boron.SetHybridization(Chem.rdchem.HybridizationType.SP2)
    try:
        embedded = AllChem.UFFOptimizeMolecule(mol, maxIters=1000)
    except:
        embedded = -1
    return mol, embedded
```

S1.2.2 GFN1-xTB and GFN2-xTB calculations

GFN1- and GFN2-xTB optimizations of neutral and charged molecules were run with the following xTB input template:

```
xtb {name}.xyz --ohess vtight --parallel 1 --gfn {version} --molden --json
  ↪ --chrg {charge} --uhf {unpaired electrons} --namespace {name} >
  ↪ {name}.xtbout.txt
```

S1.2.3 MOPAC2016 calculations

PM6 and PM7 calculations were run with the following MOPAC2016 input templates:

```
PM7 CHARGE=0 SINGLET PRECISE SUPER
name

 At1    x1  y1  z1
 At2    x2  y2  z2
 At3    x3  y3  z3
 .      .   .   .
 .      .   .   .
 .      .   .   .
```

Replacing the `PM7` keyword with `PM6` in the above example script allows a PM6 calculation.

S1.2.4 DFT calculations

DFT geometry optimization for the neutral and charged species (using the respective optimized xTB geometries as the starting geometry) was run with the following ORCA template:

```
! UKS cam-b3lyp def2-svp
# accuracy, approximations, and dispersion corrections
! tightscf rijcosX def2/j d3bj
# type of calculation
! opt
# output control
! miniprint
# calculation resources
%pal nprocs 1 end
%maxcore 2048
%base "name"
# type of input; charge; multiplicity; input
*xyzfile {charge} {multiplicity} name.xyz
```

S2 Structural descriptors and moiety distribution

To annotate the chemical library with structural information, such as the building blocks present in the molecules and the number of branching points, we utilized RDKit to search for SMARTS patterns in the molecular structures. The following table presents the SMARTS patterns used to identify the different building blocks and the presence of branching points.

Table S1: SMARTS patterns corresponding to the various aromatic building blocks

Descriptor	SMARTS
Benzene	`[#6]~1~[#6]~[#6]~[#6]~[#6]~[#6]~1`
Pyridine	`[#6]~1~[#6]~[#6]~[#7]~[#6]~[#6]~1`
Borinine	`[#5]~1~[#6]~[#6]~[#6]~[#6]~[#6]~1`
Pyrazine	`[#6]~1~[#6]~[#7]~[#6]~[#6]~[#7]~1`
1,4-diborinine	`[#5]~1~[#6]~[#6]~[#5]~[#6]~[#6]~1`
1,4-dihydro-1,4-diborinine	`[#5&H1]~1~[#6]~[#6]~[#5&H1]~[#6]~[#6]~1`
Pyrrole	`[#6]~1~[#6]~[#6]~[#7&H1]~[#6]~1`
Borole	`[#5&H1]~1~[#6]~[#6]~[#6]~[#6]~1`
Thiophene	`[#6]~1~[#6]~[#6]~[#16]~[#6]~1`
Furan	`[#6]~1~[#6]~[#6]~[#8]~[#6]~1`
Cyclobutadiene	`[#6]~1~[#6]~[#6]~[#6]~1`
Branch motif	`[*;R2r6]~1~[*;R2r6]~[*;R2r6]~[*;R2r6]~[*;R2r6]~[*;R2r6]~1`

A ratio of 10:1 in favor of benzene versus all other moieties was implemented when generating cc-hPASs (see Figure 1b in the main text for the structure generation pipeline), to ensure a more realistic distribution of cc-hPASs. In Table S2, we detail the appearance frequency of the various moieties (building blocks) across COMPAS-2x and COMPAS-2D, respectively. In this table, we provide the likelihood (frequency) of each ring type. As expected, the likelihood of a ring being benzene is approximately 10 times larger than any of the other types of building blocks across both data sets. The likelihoods of the other building blocks are approximately 10 times smaller and are very similar to one another, across both data sets. This demonstrates that the molecular generation, as well as the selection from COMPAS-2x to COMPAS-2D, were truly random.

Table S2: Distribution of the various building blocks used in COMPAS-2x and COMPAS-2D. The last two columns present the ratio of each moiety with respect to benzene.

Building Block (bblock)	COMPAS-2x	COMPAS-2D	COMPAS-2x bblock/benzene	COMPAS-2D bblock/benzene
Benzene	2148546	218582	1.000	1.000
Pyridine	218660	22181	0.101	0.101
Borinine	221475	18279	0.103	0.083
Pyrazine	209400	21243	0.097	0.097
1,4-diborinine	213398	15696	0.099	0.071
1,4-dihydro-1,4-diborinine	189897	18211	0.088	0.083
Pyrrole	217979	22178	0.101	0.101
Borole	217216	21854	0.101	0.099
Thiophene	219166	22577	0.102	0.103
Furan	216198	22222	0.100	0.101
Cyclobutadiene	214410	20253	0.099	0.092

S3 Benchmarking Semi-empirical Methods

To find a suitable semi-empirical method for high-throughput calculations, we selected a random data set of 1k cc-hPASs as our benchmark set. We optimized these molecules' geometries at the CAM-B3LYP-D3BJ/def2-SVP level of theory and calculated vibrational frequencies to ensure that the geometries were minima on the potential energy surface. For these molecules, we extracted the HOMO (highest occupied molecular orbital) and LUMO (lowest unoccupied molecular orbital) energy values.

Using the DFT-optimized geometries, we calculated the same properties with several semi-empirical methods: GFN1-xTB and GFN2-xTB,[4] as implemented in the xTB (v6.5.1.27), and PM6,[6] and PM7,[7] as implemented in the MOPAC software suite.[11] In Figure S1, we present scatter plots comparing the results of the four semi-empirical methods against the DFT values. In Table S3 we detail different evaluations of the agreement between the methods.

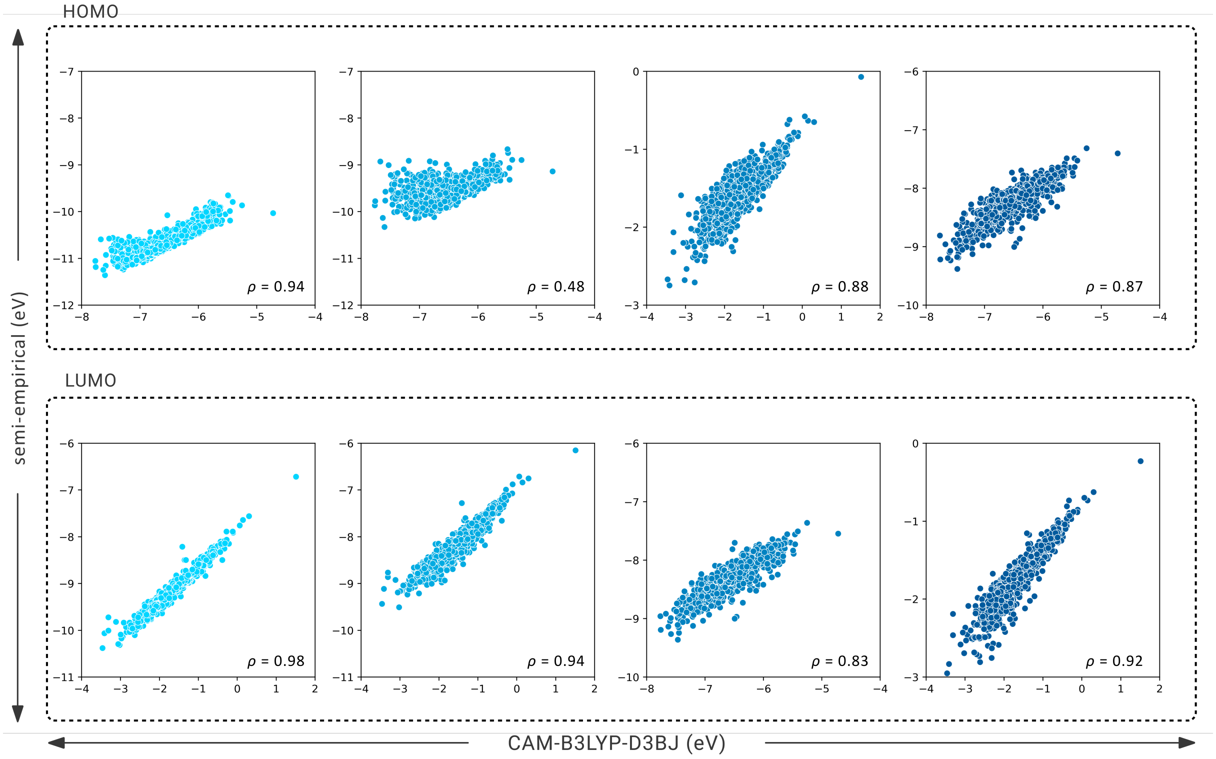

Figure S1: Benchmarking of HOMO and LUMO energies obtained with semiempirical methods against CAM-B3LYP-D3BJ/def2-SVP

All of the methods have a relatively high MAE because the value ranges are very far from the ranges of the DFT-calculated distributions. Of the two types of methods, PM6 and PM7 have markedly lower MAEs. However, since our goal is to eventually correct the semi-empirical values toward the DFT values, the linearity of the correlation (measured with the Pearson coefficient) is a more important evaluator than the MAE. In this regard, the GFN1-xTB shows the best performance for both of the properties tested. This suggests that GFN1-xTB properties may be linearly regressed better than other methods. Surprisingly, GFN2-xTB, which is a more generalized version of xTB, has a very low Pearson coefficient for the HOMO energy. In our previous investigation of cc-PBHs,[12] we found good correlations between GFN2-xTB and B3lYP values. Thus, it is likely that this discrepancy stems from the presence of the heteroatoms. However, we

Table S3: Evaluation of the correlation between various semiempirical methods and DFT values for HOMO and LUMO energies

	**GFN1-xTB**		**GFN2-xTB**		**PM6**		**PM7**	
	HOMO	LUMO	HOMO	LUMO	HOMO	LUMO	HOMO	LUMO
**R^2**	-89.998	-157.122	-49.124	-121.478	-15.890	0.590	-15.504	0.727
**RMSE**	4.084	7.488	3.031	6.590	1.759	0.380	1.739	0.310
**MAE**	4.078	7.486	3.008	6.586	1.746	0.318	1.725	0.251
**MSE**	16.686	56.073	9.191	43.433	3.097	0.145	3.026	0.096
**Pearson**	0.882	0.980	0.488	0.940	0.880	0.837	0.869	0.924
**VAR**	0.050	0.030	0.140	0.057	0.047	0.137	0.049	0.088

did not investigate this further, as it was outside the scope of the present work. PM6 and PM7 also have high Pearson coefficients (though not as high as GFN1-xTB), but these calculations do not provide total energy for the molecules. In light of this, we chose to continue with GFN1-xTB.

S4 Scatter Plots of GFN1-xTB versus DFT-Calculated Properties

In the main text, we presented contour plots of the kernel density estimates of the distributions of the GFN1-xTB data versus the CAM-B3LYP-D3BJ data. In this section, we provide scatter plots of the same data.

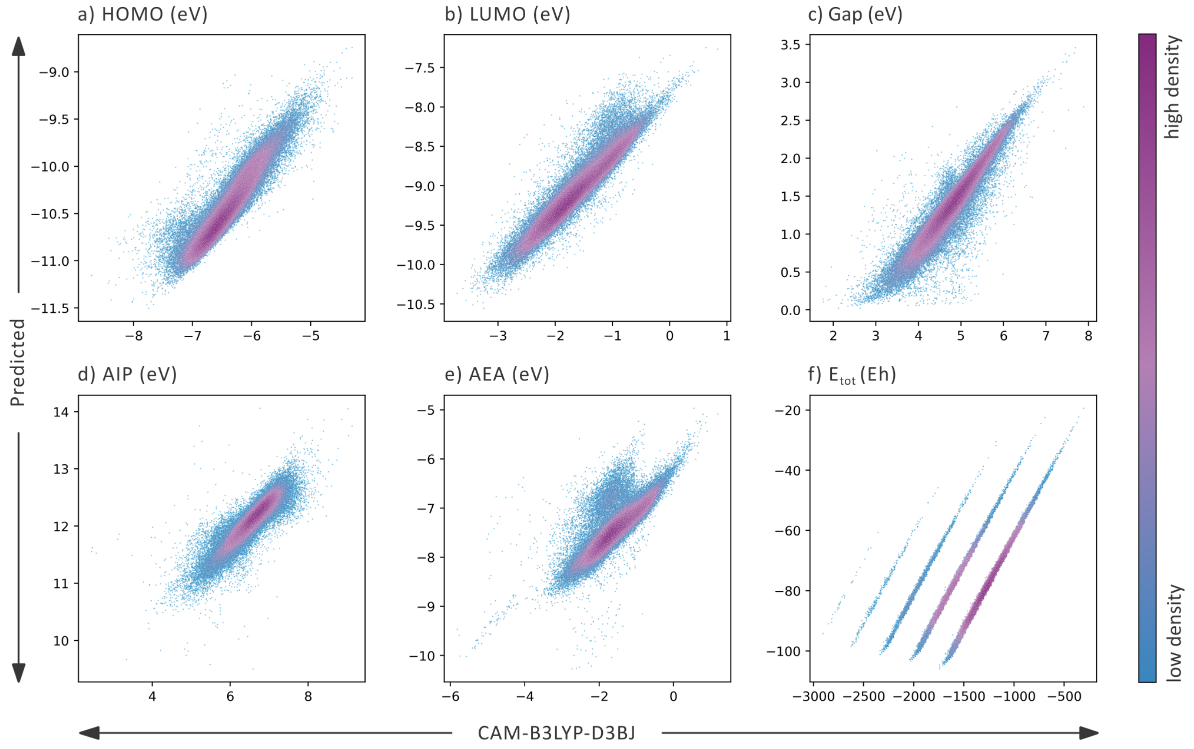

Figure S2: Comparison of electronic properties at the xTB and DFT level of theory. Each data point represents a single molecule; The DFT-calculated property values are shown on the x-axis and the GFN1-xTB-calculated property values are shown on the y-axis. The color of the data points indicates the density of points in that region: blue is low density, pink is high density.

S5 The Effect of Sulfur on E_{tot}

In this section, we discuss the unusual correlation between the E_{tot} values obtained with GFN1-xTB and CAM-B3LYP-D3BJ. As mentioned in the main text, when the E_{tot} values of the two methods were plotted in a simple scatter plot, we observed a series of highly linear correlations that appeared to have similar slopes but different intercepts (i.e., offsets). This behavior suggested that, in general, the two methods do capture the same energetic trends, but some (structural) feature is being treated differently in the two methods. Specifically, the existence of the numerous parallel groupings with similar offsets between them suggested that the feature being treated differently was appearing in different integer jumps, i.e., n × offset. By coloring the data according to the different structural features (number of atoms, number of branching points, deviation from planarity), we identified that the individual groupings were differentiated by the number of sulfur atoms in the molecule (Figure S3). Meaning, each correlation appears to be consistent only for molecules with the same number of sulfur atoms.

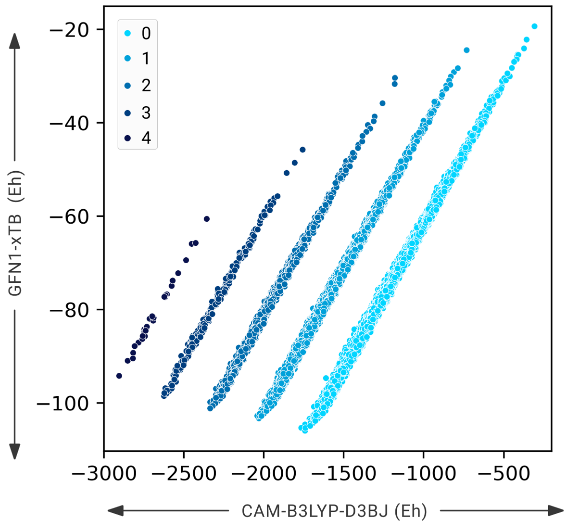

Figure S3: Scatter plot of E_{tot} calculated with CAM-B3lYP-D3BJ versus E_{tot} calculated with GFN1-xTB, for all molecules in COMPAS-2D. Each individual point represents a single molecule; the points are colored by the number of sulfur atoms.

S5.1 Linear Regression Between GFN1-xTB and CAM-B3LYP-D3BJ

To establish a correction scheme from the less expensive GFN1-xTB to the more accurate but costlier DFT level, we performed a linear regression analysis on the E_{tot} values. As detailed above, we found that the number of sulfur atoms is the major contributing variable in this correlation (Figure S4a). The resulting equation for the correction is as follows:

$$E_{\mathrm{DFT}} = 16.2132 \times E_{\mathrm{xTB}} - 343.4755 \times S + 8.7150 \tag{S1}$$

However, to maintain consistency with the other correction schemes used, we also incorporated the counts of all different types of atoms (Figure S4b). The equation for the correction is as follows:

$$\begin{aligned} E_{\mathrm{DFT}} = {} & 0.7880 \times E_{\mathrm{xTB}} - 0.2102 \times H - 36.3793 \times C - 23.6932 \times B \\ & - 395.1947 \times S - 71.4597 \times O - 52.1923 \times N + 0.1285. \end{aligned} \tag{S2}$$

As can be seen from Figure S4, both schemes provide excellent predictions of the E_{tot}, though the second one is slightly more accurate.

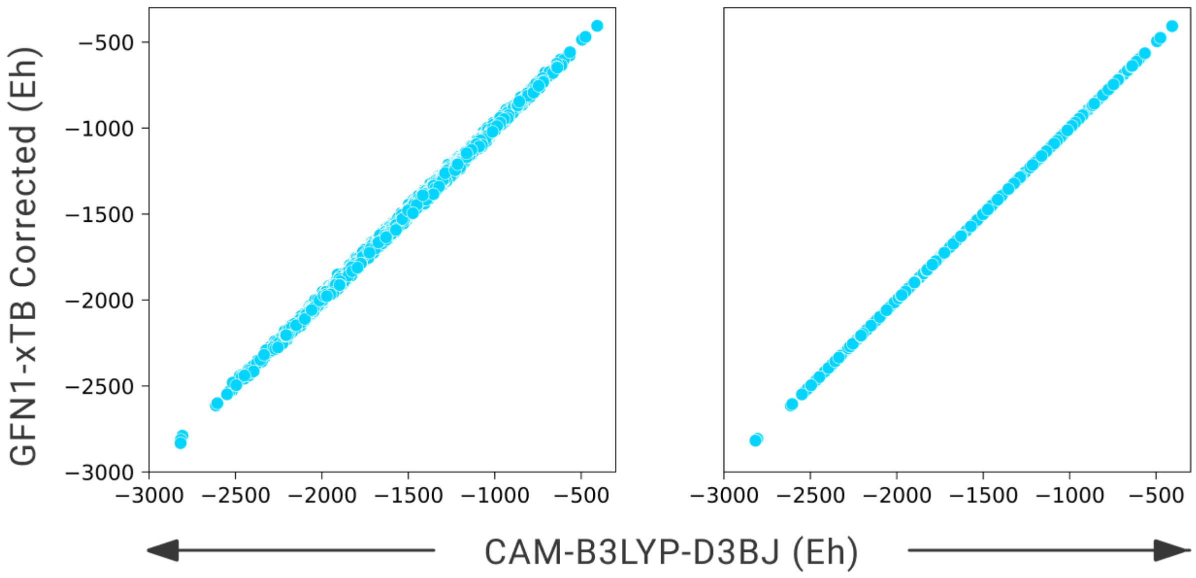

Figure S4: Scatter plots of corrected xTB E_{tot} versus DFT E_{tot}, using (a) only the number of sulfur atoms; (b) all atom counts in the molecule.

S6 Benchmarking Regression Schemes

In this section, we present the different regression schemes implemented and report the metrics of the various models used. The models were trained on a random 80/20 train-test split, using the following regression algorithms: Linear Regression, Ridge, Lasso, ElasticNet, Random Forest Regressor, K Neighbors Regressor, SVG, and ARD Regression. 5-fold cross-validation was used to optimize the parameters of the Ridge, Lasso, ElasticNet, Random Forest Regressor, and K Neighbors Regressor models as implemented in scikit-learn[13].

For each model, we tested two sets of features: a) the number of the various atoms present in the molecules and b) the number of the various building blocks. While the former is structure-agnostic (depends only on the molecular formula, not on the specific molecular structure), the latter requires knowledge of the specific cyclic building blocks that make up the molecule.

Each model+feature combination was trained to predict the different electronic properties (HOMO, LUMO, gap (HOMO-LUMO gap), AIP, and AEA) at the DFT level. We report in Table S4 the results of these experiments, which we evaluated via various metrics for each model: the average of the coefficient of determination (R^2), the average MAE, and the average RMSE computed across the five properties. We also report the residuals between the DFT and xTB values. These metrics provide insight into the accuracy and predictive power of the different correction schemes and models.

The different models used were:

1. LR - Linear Regression,
2. RLR4 - Ridge Regression (alpha = 10^{-4}),
3. RLR5 - Ridge Regression (alpha = 10^{-5}),
4. LLR4 - Lasso Regression (alpha = 10^{-4}),
5. LLR5 - Lasso Regression (alpha = 10^{-5}),
6. EN4 - Elastic Net Regression (alpha = 10^{-4}),
7. EN5 - Elastic Net Regression (alpha = 10^{-5}),
8. RF - Random Forest Regression
9. KNN1 - K-Nearest Neighbors Regression (using Bray-Curtis distance)
10. KNN2 - K-Nearest Neighbors Regression (using Canberra distance)
11. KNN3 - K-Nearest Neighbors Regression (using Cosine)
12. KNN4 - K-Nearest Neighbors Regression (using Euclidean distance)
13. KNN5 - K-Nearest Neighbors Regression (using Manhattan distance)
14. KNN6 - K-Nearest Neighbors Regression (using Minkowski distance)
15. ARD - Automatic Relevance Determination Regression,
16. SVR-RBF - Support Vector Regression with Radial Basis Function kernel.

The 'atoms' feature set refers to the models trained using the molecular formula. The 'bblocks' feature set refers to the models trained using the building block count present in the molecule.

Table S4: Error metrics for different regression models. Average R^2, RMSE, and MAE values for different regression models trained on the two feature sets.

Model	Features	MAE	PEARSON	R^2	RMSE
EN4	atoms	0.23 ± 0.21	0.94 ± 0.05	0.88 ± 0.09	0.32 ± 0.26
	bblocks	0.20 ± 0.15	0.94 ± 0.05	0.89 ± 0.09	0.37 ± 0.40
EN5	atoms	0.23 ± 0.20	0.94 ± 0.05	0.88 ± 0.09	0.31 ± 0.25
	bblocks	0.19 ± 0.13	0.94 ± 0.05	0.89 ± 0.09	0.34 ± 0.33
KNN1	atoms	3.09 ± 7.06	0.87 ± 0.06	0.76 ± 0.12	8.75 ± 20.67
	bblocks	7.37 ± 17.57	0.89 ± 0.05	0.79 ± 0.08	14.49 ± 34.80
KNN2	atoms	1.66 ± 3.59	0.89 ± 0.07	0.79 ± 0.13	4.63 ± 10.65
	bblocks	8.32 ± 19.84	0.87 ± 0.06	0.75 ± 0.10	15.63 ± 37.54
KNN3	atoms	6.66 ± 15.75	0.83 ± 0.08	0.70 ± 0.13	18.01 ± 43.30
	bblocks	12.40 ± 29.81	0.84 ± 0.05	0.70 ± 0.08	23.30 ± 56.28
KNN4	atoms	3.37 ± 7.74	0.88 ± 0.06	0.77 ± 0.11	9.50 ± 22.53
	bblocks	7.49 ± 17.86	0.89 ± 0.04	0.80 ± 0.08	14.54 ± 34.95
KNN5	atoms	3.13 ± 7.15	0.87 ± 0.06	0.76 ± 0.12	8.81 ± 20.83
	bblocks	7.37 ± 17.57	0.89 ± 0.04	0.79 ± 0.08	14.50 ± 34.82
KNN6	atoms	3.37 ± 7.74	0.88 ± 0.06	0.77 ± 0.11	9.50 ± 22.53
	bblocks	7.49 ± 17.86	0.89 ± 0.04	0.80 ± 0.08	14.54 ± 34.95
LLR4	atoms	0.23 ± 0.20	0.94 ± 0.05	0.88 ± 0.09	0.31 ± 0.25
	bblocks	0.19 ± 0.13	0.94 ± 0.05	0.89 ± 0.09	0.34 ± 0.32
LLR5	atoms	0.23 ± 0.20	0.94 ± 0.05	0.88 ± 0.09	0.31 ± 0.25
	bblocks	0.19 ± 0.13	0.94 ± 0.05	0.89 ± 0.09	0.34 ± 0.32
LR	atoms	0.13 ± 0.07	0.94 ± 0.05	0.88 ± 0.09	0.18 ± 0.10
	bblocks	0.16 ± 0.06	0.94 ± 0.05	0.89 ± 0.09	0.23 ± 0.07
RF	atoms	0.29 ± 0.38	0.94 ± 0.04	0.89 ± 0.08	0.68 ± 1.17
	bblocks	0.42 ± 0.70	0.95 ± 0.04	0.90 ± 0.07	0.87 ± 1.65
RLR4	atoms	0.13 ± 0.07	0.94 ± 0.05	0.88 ± 0.09	0.18 ± 0.10
	bblocks	0.16 ± 0.06	0.94 ± 0.05	0.89 ± 0.09	0.23 ± 0.07
RLR5	atoms	0.13 ± 0.07	0.94 ± 0.05	0.88 ± 0.09	0.18 ± 0.10
	bblocks	0.16 ± 0.06	0.94 ± 0.05	0.89 ± 0.09	0.23 ± 0.07
SVR-RBF	atoms	24.81 ± 60.44	0.89 ± 0.10	0.76 ± 0.25	42.98 ± 104.75
	bblocks	25.35 ± 61.78	0.90 ± 0.11	0.78 ± 0.26	43.09 ± 105.06

In general, it can be seen that all of the models achieve comparable results. In almost all cases, training on the building block information leads to slightly lower MAEs than training on the molecular formula. This is not surprising, as the building blocks contain more chemical information.

Thus, after evaluating the performance of the models, the Linear Regression model using xTB properties and atom counts was chosen to directly predict the DFT properties. This selection was based on its simplicity and transparency, as well as the recognition that the performance improvement achieved by other methods was not substantial enough to justify their complexity.

References

(1) Landrum, G. et al. *Greg Landrum* **2013**, *8*.

(2) Van Rossum, G.; Drake, F. L., *Python 3 Reference Manual*; CreateSpace: Scotts Valley, CA, 2009.

(3) Grimme, S.; Bannwarth, C.; Shushkov, P. *J. Chem. Theory Comput.* **2017**, *13*, 1989–2009.

(4) Bannwarth, C.; Ehlert, S.; Grimme, S. *J. Chem. Theory Comput.* **2019**, *15*, 1652–1671.

(5) Bannwarth, C.; Caldeweyher, E.; Ehlert, S.; Hansen, A.; Pracht, P.; Seibert, J.; Spicher, S.; Grimme, S. *WIREs Comput. Mol. Sci.* **2021**, *11*, e1493.

(6) Stewart, J. J. *J. Mol. Model.* **2007**, *13*, 1173–1213.

(7) Stewart, J. J. *J. Mol. Model.* **2013**, *19*, 1–32.

(8) Neese, F. *WIREs Comput. Mol. Sci.* **2012**, *2*, 73–78.

(9) Neese, F. *WIREs Comput. Mol. Sci.* **2018**, *8*, e1327.

(10) Rappé, A. K.; Casewit, C. J.; Colwell, K.; Goddard III, W. A.; Skiff, W. M. *J. Am. Chem. Soc.* **1992**, *114*, 10024–10035.

(11) Stewart, J. J. MOPAC2016, Stewart Computational Chemistry, Colorado Springs, CO, USA, Available at `http://OpenMOPAC.net`, 2016.

(12) Wahab, A.; Pfuderer, L.; Paenurk, E.; Gershoni-Poranne, R. *J. Chem. Inf. Model.* **2022**, *62*, 3704–3713.

(13) Pedregosa, F. et al. *Journal of Machine Learning Research* **2011**, *12*, 2825–2830.
